# Comprehensive Analysis of Genomic Variations in Pancreatic Adenocarcinoma by Race

**DOI:** 10.1002/cam4.71346

**Published:** 2025-11-04

**Authors:** Mohamad El Moheb, Kaelyn C. Cummins, Chengli Shen, Elio R. Bitar, Susan J. Kim, Mackenzie M. Mayhew, Hongji Zhang, Russell G. Witt, Samantha M. Ruff, Allan Tsung

**Affiliations:** ^1^ Department of Surgery University of Virginia Charlottesville Virginia USA; ^2^ School of Data Science University of Virginia Charlottesville Virginia USA

## Abstract

**Purpose:**

Racial disparities in pancreatic ductal adenocarcinoma (PDAC) incidence and outcomes persist even after adjusting for access to care and treatment received, suggesting a role for tumor‐specific molecular factors. This study aimed to compare the mutational landscape of PDAC tumor samples across racial groups using a large, diverse patient cohort.

**Methods:**

Genetic variations were analyzed using data from the AACR Project GENIE registry. Adult patients with PDAC treated at Memorial Sloan Kettering Cancer Center or Dana‐Farber Cancer Institute who underwent next‐generation sequencing were included. Somatic mutations in 680 genes were compared among White, Black, and Asian patients, controlling for multiple comparisons.

**Results:**

Among 4327 patients (mean age 66.4 years, 53% male), 61% had localized disease, while 39% had metastatic disease. The most commonly mutated genes were *KRAS, TP53, SMAD4, ARID1A, KMT2D, RNF43, CDKN2A,* and *COL7A1.* The median number of mutations per patient was 4, irrespective of race (*p*‐value = 0.45). Asian patients had significantly fewer *CDKN2A* mutations than White patients (0.7% vs. 9.1%, *p* = 1.9 × 10^−5^) and Black patients (0.7% vs. 8.1%, *p* = 7.4 × 10^−4^) as well as fewer cell‐cycle‐related mutations (4.4% vs. 11.5% and 13.0%, respectively; *p* = 0.012). No significant racial differences were observed in tumor mutational burden (TMB), microsatellite instability, or clinically targetable mutations (*p*‐values > 0.05).

**Conclusions:**

Asian patients with PDAC had fewer CDKN2A mutations, a genotype linked to improved survival and reduced tumor aggressiveness. Genomic profiles were otherwise similar across races, suggesting that disparities are likely driven by non‐genomic factors beyond DNA sequence alterations, such as epigenetic modifications or posttranslational changes. Integrating multiomic and clinical data is crucial to better understand and address these disparities.

## 1 Background

Pancreatic cancer is the third leading cause of cancer mortality in the US, with an average 5‐year survival rate of only 13% [1]. Well‐documented racial disparities exist in both incidence and treatment outcomes [2, 3, 4]. While socioeconomic status and rurality influence access to care [5], racial differences in outcomes persist even after adjusting for these factors and treatment received [6]. Asian patients have shown improved survival, even after controlling for clinical variables and treatment modalities, while Black patients consistently demonstrate worse outcomes compared to White patients [7]. These findings raise important questions about the role of tumor biology in shaping outcomes across racial groups, in addition to social and systemic determinants.

Understanding genomic variations by race in pancreatic ductal adenocarcinoma (PDAC) is critical, as tumor genetics significantly influence disease progression, treatment response, and survival [8]. While tumor genomic profiles are known to vary across racial groups due to differences in environmental exposure and genetic ancestry [9, 10], there is limited high‐quality evidence comparing the mutational profile of pancreatic cancer across races. Prior genome association studies, including those examining actionable mutations have predominantly focused on White cohorts [11]. This underrepresentation of minorities limits our understanding of how ancestry influences incidence, prognosis, and potential disparities in a diverse patient population. Furthermore, existing research has primarily focused on driver mutations without providing comprehensive genomic analysis of actionable mutations, tumor mutational burden (TMB), and microsatellite instability (MSI)—factors that could provide crucial insights into the biological mechanisms underlying racial disparities in pancreatic cancer outcomes [12].

This study aims to address these gaps by systematically exploring the somatic mutational landscape of PDAC across racial groups using a large, diverse patient cohort. By elucidating genomic variations by race, we seek to improve our understanding of biological factors that may influence outcomes in PDAC, which may inform future personalized therapeutic strategies.

## 2 Methods

This study was deemed exempt from informed consent by the institutional review board of the University of Virginia due to the publicly available and deidentified nature of the dataset.

### 2.1 Study Population and Data Source

We analyzed genetic variations from next‐generation sequenced (NGS) tumor samples of patients with histologically confirmed pancreatic adenocarcinoma treated at either Memorial Sloan Kettering Cancer Center (MSKCC) or Dana‐Farber Cancer Institute (DFCI) between 2013 and 2024. Clinical and genomic data were extracted from the American Association for Cancer Research Project Genomics Evidence Neoplasia Information Exchange (GENIE) registry (version 16.1). Patients with primary or metastatic pancreatic ductal adenocarcinoma (PDAC) were included, while those with unknown tumor sample type or multiple sample types lacking site‐specific annotation were excluded from the analysis.

Our investigation followed a systematic approach to characterize racial differences in the genomic landscape of pancreatic cancer. We conducted five main analyses: (1) evaluation of commonly mutated genes, (2) assessment of tumor mutational burden, (3) examination of microsatellite instability, (4) assessment of clinically targetable mutations, and (5) analysis of key biological pathways. For each analysis, we first compared findings across self‐reported race (White, Black, and Asian) in the overall cohort, followed by stratified analyses by tumor sample type (primary versus metastatic) to identify potential stage‐specific differences.

### 2.2 Genomic Analysis

MSKCC and DFCI contributed NGS data from a total of seven different panels, the specifics of which have been previously described [13]. We analyzed sequence variations across 680 genes, comparing profiles between racial groups and tumor stages. Our analysis focused on non‐silent mutations, specifically including frame shift deletions and insertions, in‐frame deletions and insertions, missense mutations, nonsense mutations, nonstop mutations, splice site alterations, and translation start site mutations. For the most commonly mutated genes analysis, we identified the smallest set of genes that, when considered together, were mutated in over 97% of the patient cohort. This resulted in the selection of the eight most frequently mutated genes. This approach balances the need for sensitivity while minimizing false negative results due to multiple comparison correction.

### 2.3 Tumor Mutational Burden and Microsatellite Instability

TMB was treated as a continuous variable which corresponds to the number of mutations per megabase pair sequenced. TMB was stratified by sample type and then compared across race groups. MSI status was determined by analyzing mutations in the primary mismatch repair (MMR) genes: MLH1, MSH2, MSH6, PMS2, and EPCAM.

### 2.4 Clinically Actionable Mutations

Clinically targetable genes were identified using the OncoKB database, which is prospectively maintained by MSKCC [14]. We restricted our analysis to Level 1–3 therapeutic targets, which correspond to FDA‐approved drugs, standard care drugs, and drugs with demonstrated clinical benefit (Table S1). Level 4 genes, which have only biological evidence without clinical validation, were excluded. Although MSI status and high TMB are clinically actionable in practice, they were analyzed separately from other targetable mutations in this study [15].

### 2.5 Pathway Analysis

We examined genomic alterations in key biological pathways based on existing literature [16]. The complete mapping of genes to their respective biological pathways is detailed in Table S2.

### 2.6 Statistical Analysis

Mutational frequencies were calculated for each gene and compared across racial groups. To ensure accurate frequency calculations when different sequencing panels were used, we normalized the frequencies by using the number of patients who had sequencing data available for each specific gene as the denominator, rather than the total study population. Comparisons across racial groups were performed using Chi‐square or Fisher's exact test, where appropriate. The Benjamini–Hochberg method was used to correct for multiple testing, with a threshold of *p* < 0.05 being used for statistical significance. Statistical analysis was performed using R, version 4.3.1 (R Project for Statistical Computing).

## 3 Results

A total of 4327 patients were included, 67% of whom were treated at MSKCC, while 33% were treated at DFCI. The average age was 66.4 (SD: 10.2) years, and 53% were male. Among those studied, 61% (90% White, 4% Black, 6% Asian) had primary disease, and 39% (90% White, 5% Black, 5% Asian) had metastatic disease.

**FIGURE 1:**
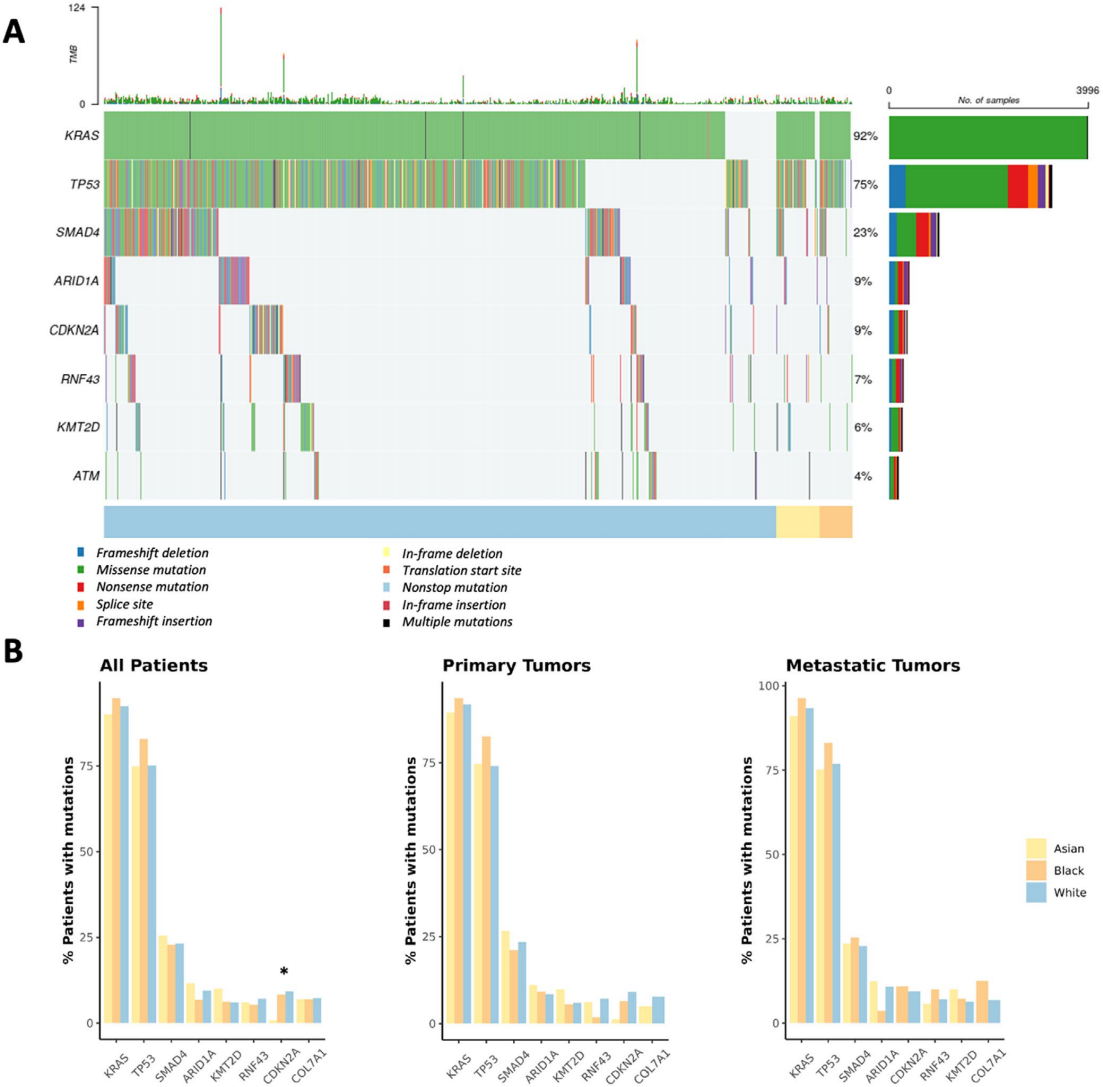
Mutation landscape of the eight most commonly mutated genes. (A) Oncoplot illustrating the mutational profile of all patients for the most commonly mutated genes. (B) Bar plots showing the frequency of these mutated genes across racial groups in all patients and further stratified by tumor subtype.

The eight most frequently mutated genes across all racial groups were KRAS, TP53, SMAD4, ARID1A, KMT2D, RNF43, CDKN2A, and COL7A1 (Figure 1). Asian patients had significantly lower mutation rates in the CDKN2A gene compared to both Black (0.7% vs. 8.1%, *p* = 7.4e‐4) and White patients (0.7% vs. 9.1%, *p* = 1.9e‐5). After stratifying by tumor sample type, this difference persisted between Asian and White patients with primary tumors (1.2% vs. 9.1%, *p* = 0.002). However, it was not significant between Asian and Black patients with primary tumors (*p* = 0.33) or among patients with metastatic disease regardless of race (*p* = 1). While Black patients exhibited a higher frequency of TP53 mutations compared to White patients (82.8% vs. 74.7%), this difference did not reach statistical significance after adjusting for multiple comparisons (*p* = 0.089).

The median number of mutations per patient was consistent across racial groups (median 4, *p* = 0.45). Similarly, the median TMB was also the same across racial groups (Table 1).

**TABLE 1:** Tumor mutational burden in pancreatic cancer tumor samples by race, for all patients and stratified by sample type.

	Median TMB	*p*
Overall		
Asian (*n* = 251)	1.72 [1.20, 2.58]	0.66
Black (*n* = 192)	1.72 [1.29, 2.59]	
White (*n* = 3884)	1.89 [1.29, 2.65]	
Primary tumor		
Asian (*n* = 162)	1.72 [1.20, 2.39]	0.47
Black (*n* = 109)	1.72 [1.20, 2.39]	
White (*n* = 2362)	1.72 [1.20, 2.58]	
Metastatic tumor		
Asian (*n* = 89)	2.11 [1.20, 2.79]	0.67
Black (*n* = 83)	2.11 [1.40, 2.82]	
White (*n* = 1522)	1.99 [1.29, 2.86]	

*Note: p*‐values were calculated using the Kruskal–Wallis test.

Abbreviation: TMB, tumor mutational burden.

Analysis of MMR genes also revealed no significant differences in mutation rates among racial groups, both overall and when stratified by tumor type, with less than 4% of patients exhibiting mutations in mismatch repair genes (Table 2).

**TABLE 2:** Mutations in mismatch repair and actionable genes in pancreatic cancer tumor samples by race, for all patients and stratified by sample type.

	MMR mutations	*p*	Actionable mutations	*p*
Overall				
Asian (*n* = 251)	5 (2.0%)	0.32	30 (12.0%)	0.72
Black (*n* = 192)	5 (2.6%)		28 (14.6%)	
White (*n* = 3884)	140 (3.6%)		511 (13.2%)	
Primary tumors				
Asian (*n* = 162)	4 (2.5%)	0.56	17 (10.5)	0.71
Black (*n* = 109)	3 (2.8%)		15 (13.8%)	
White (*n* = 2362)	92 (3.9%)		286 (12.1%)	
Metastatic tumors				
Asian (*n* = 89)	1 (1.1%)	0.52	13 (14.6%)	0.98
Black (*n* = 83)	2 (2.4%)		13 (15.7%)	
White (*n* = 1522)	48 (3.2%)		225 (14.8%)	

*Note: p*‐values were calculated using the Chi‐square test or Fisher's exact test when expected cell counts were < 5.

Abbreviation: MMR, mismatch repair genes.

Thirteen genes were considered potentially clinically targetable based on the OncoKB database (“BRCA1”, “BRCA2”, “ERBB2”, “MTAP”, “NRG1”, “NTRK1”, “NTRK2”, “NTRK3”, “PALB2”, KRAS, PALB2, TP53, BRAF). 12%–15% of patients had at least one mutation that is potentially clinically targetable; however, there was no statistically significant difference in the rate of mutations across groups (Table 2).

**FIGURE 2:**
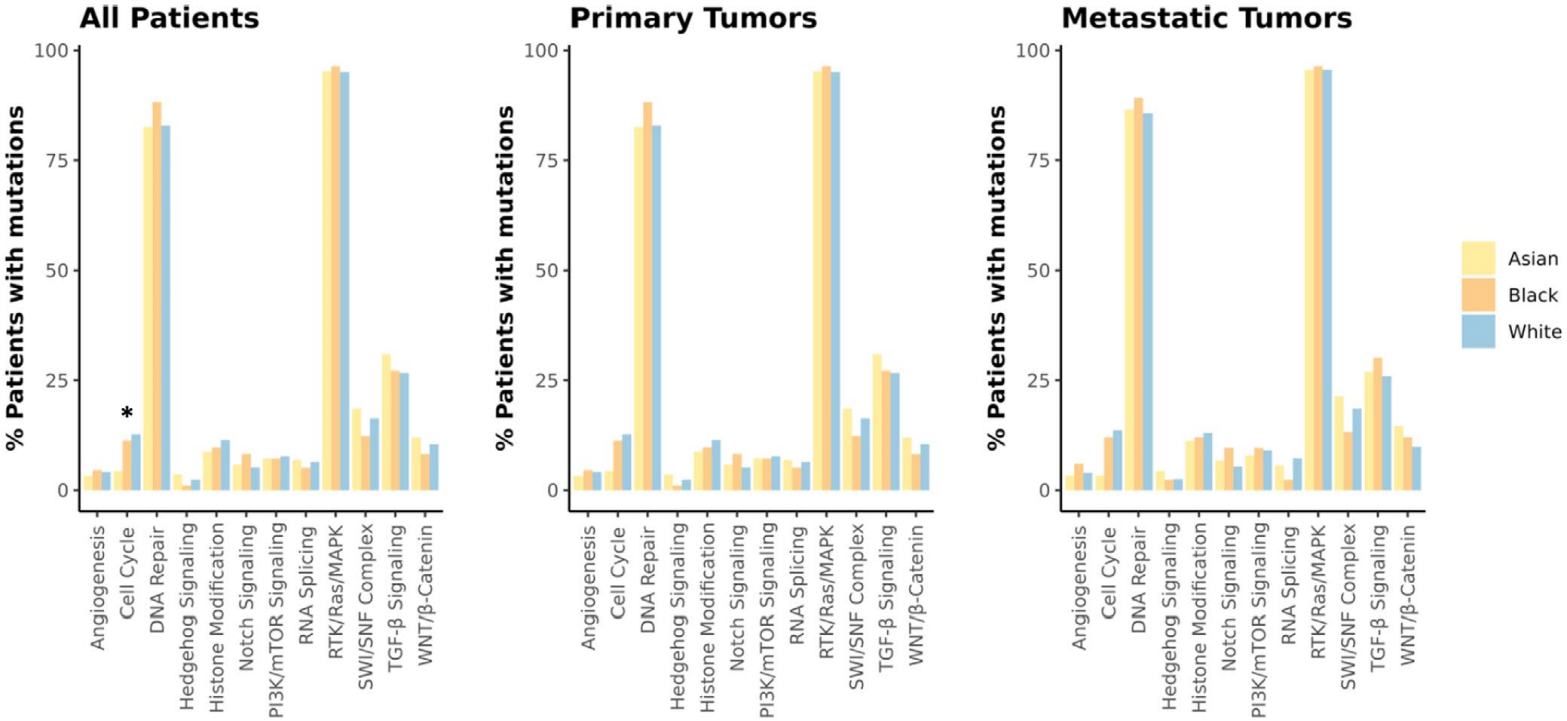
Mutation frequencies across pathways by race in all patients and stratified by tumor type. Asterisk depicts statistically significant results adjusting for multiple comparisons.

When comparing mutations in key biologic pathways, the pathways that were the most frequently mutated were RTK/RAS/MAPK, DNA repair pathway, and TGF‐beta. Asian patients also had significantly fewer mutations in cell‐cycle‐related genes compared to White and Black patients (4.4% vs. 11.5% and 13.0%, respectively; *p* = 0.012) (Figure 2). However, this association lost statistical significance when analyses were stratified by tumor sample type.

## 4 Discussion

This study represents the largest and most comprehensive analysis to date of the genomic profile of pancreatic cancer across racial groups. Our results demonstrated a significantly lower rate of CDKN2A mutations among Asian patients compared to White and Black patients. However, this difference did not persist in all subgroup analyses, particularly between Asian and Black patients with primary tumors. This might be due to limited statistical power within smaller racial subgroups, which may have reduced our ability to detect modest differences. This finding was further reflected in the overall lower rate of cell cycle gene mutations in Asian patients, primarily driven by the lower CDKN2A mutation frequency. CDKN2A encodes the tumor suppressor protein p16, which, when mutated, leads to unchecked cell cycle progression and has been associated with early tumor progression and poor clinical outcomes in PDAC [17]. Interestingly, CDKN2A‐inactivated pancreatic cancer exhibits increased sensitivity to paclitaxel and irinotecan, with paclitaxel in particular mimicking the effects of CDKN2A restoration [18]. This is linked to improved therapeutic outcomes in these patients [18]. These findings could provide a crucial link to understanding the observed survival disparities in pancreatic cancer where Asian patients consistently demonstrate better outcomes. Analysis of the National Cancer Database (2010–2019) revealed a median survival of 11.3 months for Asian American, Native Hawaiian, and Pacific Islander patients, compared to 8.9 months for Caucasians and 8.1 months for African Americans (*p* < 0.0001) [7]. This difference persisted on multivariable analysis even after adjusting for sociodemographics and treatment received, with Indian, Chinese, Korean, and Vietnamese subpopulations having a decreased mortality risk compared to White patients [7]. Similar findings were observed in the Kaiser Permanente Southern California integrated health system, where Asians showed improved survival compared to non‐Hispanic Whites [19]. Given the established role of CDKN2A loss in driving poor outcomes, its lower frequency in Asian patients may, at least in part, explain their relatively improved survival, especially when other clinical and patient‐related factors are accounted for.

Although not statistically significant, we observed a trend towards a higher rate of TP53 mutations in Black patients. This finding is consistent with observations in other cancers [20]. Goel et al. [20] found that TP53 mutations were more frequent in Black women with breast cancer (62%) compared to White women (37%). In gastric cancer, van Beek et al. showed a significantly higher rate of TP53 mutations in African American patients compared to Caucasian and Asian patients (89% vs. 40% and 56% respectively). These findings could have therapeutic implications, as recent clinical trial data suggest that patients with TP53 mutations may have an improved response to adjuvant gemcitabine. Specifically, post hoc analysis of the CONKO‐001 trial demonstrated that TP53 mutation was a positive predictive factor for gemcitabine efficacy [21]. As such, appropriate application of precision chemotherapy in these patients may, therefore, help close the racial outcome gap.

Apart from these findings, the genomic profiles were largely similar across racial groups, with the most frequently mutated genes showing consistent patterns. This uniformity contrasts with other malignancies where significant racial differences in genomic alterations are well‐documented. For instance, in breast cancer, PIK3CA mutations occur more frequently in White women compared to Black women [22, 23]. Prostate cancer exhibits even more pronounced racial variations: Black men show higher frequencies of SPOP and ZFHX3 mutations but lower rates of TMPRSS2‐ERG fusions and PTEN deletions compared to White men [24, 25], while Asian men demonstrate increased FOXA1 mutations and fewer PTEN alterations [26, 27]. The lack of difference in TMB across racial groups in pancreatic cancer also differs from patterns observed in other malignancies. Head and neck cancer, non‐small cell lung cancer, and prostate cancer all demonstrate higher TMB in Black patients compared to White and Asian patients [28, 29, 30]. In contrast, we found MSI to be uniformly low across all racial groups in our pancreatic cancer cohort, which aligns with findings in other cancer types where racial differences in MSI rates are generally less pronounced [31].

The lack of noticeable genomic differences across racial groups suggests that the sources of disparities in pancreatic cancer outcomes may not be primarily genomic in origin. Other molecular factors, including the tumor microenvironment and metabolic pathways, could be involved, explaining the persistent difference in outcomes even after adjusting for treatment received and socioeconomic status. For instance, studies have shown that the tumor microenvironment, including the immune cell infiltrate and stromal components, can significantly influence tumor progression and treatment response. In pancreatic cancer, a dense desmoplastic stroma has been shown to promote tumor growth and resistance to therapy [32, 33]. While racial differences in the tumor microenvironment of pancreatic cancer are not yet fully characterized, studies in other cancers, like breast cancer, have shown that Black women tend to have a higher density of tumor‐associated macrophages, which are associated with a poorer prognosis [34, 35]. Furthermore, metabolic reprogramming is a hallmark of cancer, and differences in metabolic pathways have been observed across racial groups [36]. These molecular differences may also manifest in transcriptional differences, proteomic level changes, posttranslational modifications, or sequence variations that future studies should explore. A recent study performed a transcriptomic analysis on 28 patients with pancreatic cancer comparing Black and White patients revealed differential expression in 1200 genes between the two groups, impacting over 40 canonical pathways [37].

This study has limitations. First, we did not analyze structural variations (e.g., deletions, amplifications, rearrangements) due to the high rate of missing data for such analyses. Second, while the AACR Project GENIE database is a valuable resource, it primarily includes data from major cancer centers. Specifically, our cohort was only composed of patients treated at MSKCC and DFCI, which may not be fully representative of the broader population of pancreatic cancer patients in the US, potentially introducing selection bias. Third, the GENIE database, while rich in genomic data, has limited clinical information (e.g., treatment histories, response to therapy, environmental exposures), limiting our ability to fully explore the relationship between genomic alterations and clinical outcomes. Additionally, it lacks information on whether tumor samples were obtained at diagnosis or at different stages of treatment, which may influence the mutational landscape observed. Fourth, our study may have been limited by statistical power, especially within certain underrepresented racial subgroups. With our cohort being predominantly composed of White patients (90%), the smaller sample sizes for Black and Asian patients may have reduced our ability to detect true differences in mutation frequencies. For example, although TP53 mutations were more frequent in Black patients compared to White patients, this difference did not reach statistical significance, possibly due to insufficient sample size within subgroups. Lastly, our analysis focused on mutations in the coding regions of a set of 680 genes. However, noncoding regions of the genome, such as regulatory elements, can also play a significant role in cancer development and progression [38, 39], and restricting the analysis to these pre‐selected genes may have limited the discovery of novel associations.

## 5 Conclusions

This study revealed a significantly lower rate of CDKN2A mutations among Asian patients, which may explain the improved survival seen in this patient population even after accounting for access to care and treatment received. Beyond this distinction, the genomic profiles were remarkably similar across racial groups, including TMB and MSI status, contrasting with the pronounced genomic differences observed in other malignancies. This genetic homogeneity suggests that persistent racial disparities in pancreatic cancer outcomes likely stem from factors beyond tumor genomics, including differences in epigenetic modifications, posttranslational regulation, tumor microenvironment interactions, metabolic pathways, and host immune responses. Future research integrating multiomic data with clinical information will be crucial for understanding these complex disparities and developing effective strategies to improve outcomes for all patients with pancreatic cancer.

## Author Contributions

**Mohamad El Moheb:** conceptualization, investigation, methodology, formal analysis, writing – original draft, visualization. **Kaelyn C. Cummins:** writing – original draft. **Chengli Shen:** project administration, data curation, methodology, formal analysis. **Elio R. Bitar:** writing – review and editing, writing – original draft. **Susan J. Kim:** writing – review and editing, visualization, data curation. **Mackenzie M. Mayhew:** writing – review and editing, data curation. **Hongji Zhang:** writing – review and editing. **Russell G. Witt:** writing – review and editing, methodology. **Samantha M. Ruff:** supervision, resources, investigation, writing – review and editing, methodology. **Allan Tsung:** conceptualization, investigation, writing – review and editing, supervision, resources, methodology.

## Conflicts of Interest

The authors declare no conflicts of interest.

## Supporting information


**Table S1:** Actionable genes.
**Table S2:** Key biologic pathways and their associated genes.

## Data Availability

The genomic and clinical data analyzed in this study were obtained from the publicly available AACR Project GENIE registry. All data are deidentified and can be accessed at https://www.aacr.org/professionals/research/aacr‐project‐genie/. No additional patient‐level data are available from the authors.
