# Supplementary material for: Comprehensive Analysis of Genomic Variations in Pancreatic Adenocarcinoma by Race
Source: Cancer Med. 2025 Nov 4;14(21):e71346. doi: 10.1002/cam4.71346 (PMC12585320; doi:10.1002/cam4.71346)
Supplement: Supplementary file 1 — Table S1: Actionable genes. Table S2: Key biologic pathways and their associated genes. [file CAM4-14-e71346-s001.docx]

**SUPPLEMENTARY MATERIAL**

**Supplementary Table 1. Actionable Genes**

| **Gene** |
| --- |
| **BRCA1** |
| **BRCA2** |
| **ERBB2 (HER2)** |
| **MTAP** |
| **NRG1** |
| **NTRK1** |
| **NTRK2** |
| **NTRK3** |
| **PALB2** |
| **KRAS** |
| **BRAF** |
| **TP53** |

**Supplementary Table 2. Key Biologic Pathways and Their Associated Genes**

| **Biologic Pathway** | **Genes** |
| --- | --- |
| **RTK/Ras/MAPK activation** | KRAS, GNAS, MAP2K4, BRAF, EGFR, FGFR2, ERBB2, MET, ERBB3, FGFR1, RAF1, ALK, RET, NTRK1, ERBB4, FGFR3, MAP3K13, MAP3K1, MAP3KI, MAP3K6, MAP2K1, FGFR3, FGFR4, ERBB3, NRAS, HRAS, NTRK2, ROS1 |
| **DNA damage repair** | TP53, STK11, MLH1, MLH2, PMS2, MSH6, POLE, POLD1, TP53BP1, CDK12, BRCA2, BRCA1, ATM, CHEK2, PALB2, FANCF, RAD54L, FANCC, MRE11A, FANCG, RAD51C, RAD51L3, RAD54L, FANCL, FANCM, FANCA, FANCD2, RAD50, FANCI, RAD51, XRCC3, ERCC4, FANCE, CHEK1, BARD1, BRIP1, ATR |
| **Cell cycle control** | CDKN2A, CDKN2B, RB1, CDK4, CDK6, CCND1, CCND2, CCND3, CCNE1, CDKN1B, CDK8, CDKN2C |
| **Transforming growth factor-beta signaling** | SMAD4, TGFBR2, SMAD2, SMAD3, ACVR1B |
| **Histone modification** | KDM6A, KDM5A, KDM5C, MLL2, MLL3, MLL, EP300, SETD2, BAP1, ASXL1, MYST3, CREBBP |
| **SWI/SNF protein complex** | ARID1A, ARID1B, ARID2, PBRM1, SMARCA4, SMARCB1, SMARCD1 |
| **PI3K/mTOR signaling** | PIK3CA, PTEN, PIK3R1, TSC2, TSC1, MTOR, AKT1, AKT2, AKT3 |
| **WNT/beta-catenin pathway** | RNF43, CTNNB1, APC, LEF1, AXIN1, TCF3 |
| **RNA splicing** | RBM10, SF3B1, U2AF1, QKI |
| **Notch pathway** | NOTCH1, NOTCH2, NOTCH3, NOTCH4 |
| **Angiogenesis** | VEGFA, VHL, FLT1, FLT3, FLT4 |
| **Hedgehog signaling** | SMO, PTCH1, GLI1 |
